# Social protection and the International Monetary Fund: promise versus performance

**DOI:** 10.1186/s12992-024-01045-9

**Published:** 2024-05-08

**Authors:** Alexandros Kentikelenis, Thomas Stubbs

**Affiliations:** 1 Department of Social and Political Sciences, Bocconi University, Milan, Italy; 2 Department of Politics and International Relations, Royal Holloway, University of London, London, UK

**Keywords:** Social protection, Austerity, International Monetary Fund

## Abstract

**Background:**

Countries in the Global South are currently facing momentous economic and social challenges, including major debt service problems. As in previous periods of global financial instability, a growing number of countries have turned to the International Monetary Fund (IMF) for financial assistance. The organization has a long track-record of advocating for extensive fiscal consolidation—commonly known as ‘austerity’—for its borrowers. However, in recent years, the IMF has announced major initiatives for ensuring that its loans support social spending, thus aiding countries in meeting their development targets and the Sustainable Development Goals. To assess this track record, we collected spending data on 21 loans signed in the 2020–2022 period, including from all their periodic reviews up to August 2023.

**Results:**

We find that austerity measures remain a core part of the organization’s mandated policies for its borrowers: 15 of the 21 countries studied here experience a decrease in fiscal space over the course of their IMF programs. Against this fiscal backdrop, social spending floors have failed to live up to their promise. There is no streamlined definition of these floors, thus rendering their application haphazard and inconsistent. But even on their own terms, these floors lack ambition: they often do not foresee trajectories of meaningful social spending increases over time, and, when they do, many of these gains are eaten up by soaring inflation. In addition, a third of social spending floors are not implemented—a much lower implementation rate from that for austerity conditions, which the IMF prioritizes. In several instances, where floors are implemented, they are not meaningfully exceeded, thus—in practice—acting as social spending ceilings.

**Conclusions:**

The IMF’s lending programs are still heavily focused on austerity, and its strategy on social spending has not represented the sea-change that the organization advertised. At best, social spending floors act as damage control for the painful budget cuts: they are instruments of social amelioration, underpinned by principles of targeted assistance for highly disadvantaged groups. Alternative approaches rooted in principles of universalism can be employed to build up durable and resilient social protection systems.

## Background

Since the emergence of the COVID-19 pandemic, countries in the Global South have not only had to deal with the pressing health emergency but also with a highly destabilizing international economic environment [1]. Economic slowdowns stemming from pandemic-abatement policies were followed by fiscal crises as a result of rising energy and food prices. The interest rate increases by central banks in the Global North compounded these pressures, leading to increases in debt service requirements for low- and middle-income countries [2, 3]. By end-2023, 36 low- and lower-middle income countries were in debt distress or at high risk of it, and many more have brewing debt concerns [4]. This economic situation has alarming implications for health.

**Fig. 1:**
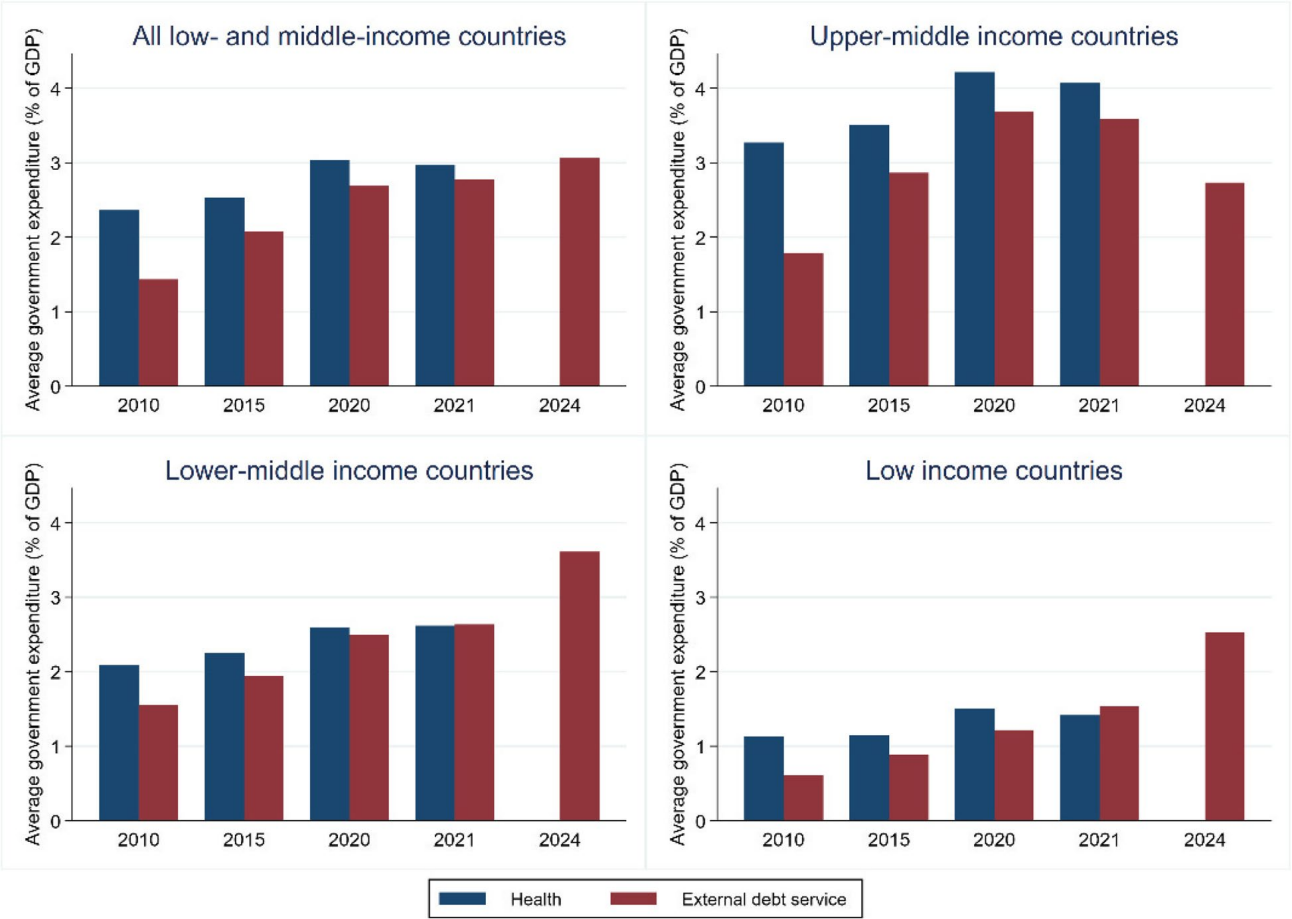
Government expenditure on health and external debt service as a share of GDP in low, lower-middle, upper-middle, and high income countries. Note: Debt service includes public and publicly guaranteed external debt but excludes International Monetary Fund debt service (charges and repurchases) and domestic debt. Data sources: [5, 6]

As shown in Fig. 1, external debt service is anticipated to be over 3% of GDP in 2024, thus exceeding public spending on health at the height of the pandemic in 2020. When disaggregated into income groups, upper-middle income countries exhibited debt service costs of 3.7% of GDP in 2020 but this decreased to 2.7% by 2024, whereas lower-middle income countries experienced a rapid escalation of these costs, increasing from 2.5% of GDP in 2020 to 3.6% by 2024. The debt servicing costs of low income countries are rapidly increasing too, more than doubling as a share of GDP, from 1.2% in 2020 to 2.5% in 2024. Even though it appears that debt service problems are less pronounced for low income countries, this is because they are de facto shut out of private markets, thus elevating the importance of the IMF to them as a key source of financing. Health spending data is not available after 2021, but it is likely that as the acute phase of the pandemic abated, health spending correspondingly declined across all income groups.

Against the backdrop of intense fiscal and debt pressures and mounting social dislocations, Global South countries have increasingly turned to the International Monetary Fund (IMF), the world’s lender of last resort, for financial assistance [7]. The organization makes such funding contingent on the borrowing governments introducing a range of policy reforms, known as conditionality, that commonly target fiscal policy, taxation, and a broad swath of development policies. In particular, the IMF’s budget targets are devised in quarterly or biannual program reviews in light of the most up-to-date estimates of prospective output, inflation, and available external financing. Such forecasts—like any economic projection—are not a precise science, but they do factor in all available information on expected revenues, expenditures, and inflation. To ensure implementation of the mandated targets and reforms, the IMF assesses performance during these reviews and disburses its loans in ‘tranches’: successful implementation of the mandated reforms unlocks a new batch of financing, while inadequate progress stalls the disbursement of funds [8] Excluding emergency pandemic-era assistance, 47 countries turned to the IMF for conditionality-carrying loans between 2020 and 2023. Of these, the majority were for three- or four-year lending programs (the Extended Credit Facility or Extended Fund Facility) that stipulate extensive policy reforms and have a longer timeframe for their implementation. The remainder were shorter, more targeted lending programs, commonly lasting one year.

The IMF’s increasing involvement in developing countries over the medium term brings to the fore long-standing questions on the social and health consequences of the organization’s policy advice, debates dating back to the 1980s when pioneering UNICEF research pointed to the human costs of economic reform policies [9]. Since then, voluminous scholarship has documented how IMF-mandated austerity measures undermine public health. This literature has pointed to various channels through which this relationship operates. First, and most conspicuously, the IMF’s reforms stipulate extensive budget cuts, which in turn directly affect the availability and quality of a range of social and health policies [10–17]. Second, these same reforms have also indirect implications for social policies, as they are associated with overall economic contractions and thus limit the available public resources to be invested into social protection over the medium-term [18]. Finally, austerity policies have manifold implications for the social determinants of health—that is, the conditions in which people are born, age, and work in [19]. For example, IMF programs are associated with steep increases in inequalities [20–22]—especially linked to adverse effects on children and women [23, 24]—and with decreasing eligibility for or access to social protection policies [10], developments which in turn increase individual health risks and have long-lasting consequences for health status.

Partly in response to such criticisms, the IMF now portends to have reformed its practices. In recent years, the IMF introduced new policies and strategies that are intended to protect and even increase social expenditures in order to ensure that its conditions do not hamper the ability of countries to invest in social protection and meet the Sustainable Development Goals [25–32]. The most comprehensive review to the IMF’s modus operandi vis-á-vis social protection came in June 2019, when the organization launched its *Strategy for IMF Engagement on Social Spending* [29] outlining the institutional view on social spending that will guide IMF staff on social protection, health, and education for the foreseeable future. According to former Managing Director Christine Lagarde, the strategy ‘is consistent with and supportive of the scope and objectives of social protection as defined by the international community, notably in the SDGs’ [27]. The strategy seeks to link IMF lending practices to social spending considerations, provided that this still foregrounds efficiency and fiscal sustainability considerations, and emphasizes the need to mitigate the adverse consequences of austerity and related structural reforms on vulnerable populations.

The key operational instrument in the IMF’s new strategy were the so-called ‘social spending floors’—quantitative targets that spell out the minimum public expenditure on selected social policies for countries under IMF programs. These floors are not, in actuality, a novel practice. They have been increasingly introduced in IMF programs since the turn of the millennium. As recently as 2019, four out of five IMF programs included at least one social spending floor [10]. Instead, the new strategy formalized such engagement and sought to clearly operationalize it for its staff involved in the design of lending programs. The benchmark for success was to be whether ‘on average, education and health spending in program countries either increased by more than, or at the same rate as, spending in non-program countries’ [29].

As a starting point, this type of comparison is problematic on two counts. First, it suggests countries that turn to the IMF for support are comparable to those that do not. For example, low-income countries that do not borrow from the IMF may face major developmental challenges—like the incidence of war—which depress social expenditures. Second, the comparison implies that comparing spending data of the year after IMF loan approval with that of the year before is a reasonable approach. However, in the year prior to entering an IMF agreement, countries tend to face major economic crises, which generally depress social spending. Assuming that the IMF helps stabilize economic conditions, a social spending increase may signal a return to a longer-term spending trend, rather than be attributable to the IMF program.

Even with these shortcomings, the IMF’s new strategy on social spending can represent a step forward for the organization, if it succeeds. This would mean that the IMF has genuinely sought to revamp its practices, albeit within the constraints of its own bureaucratic approach to this issue. In contrast, it is also possible that the IMF can use this new strategy to attempt to placate critics by only ceremonially changing underlying practices to comply with the new operational guidance [33].

This article seeks to take stock of these empirical issues by offering extensive new data to examine the IMF’s post-pandemic performance vis-á-vis social protection. As we have seen, IMF programs are designed after the organization’s staff model expected fiscal needs and inflationary pressures. This means that if we actually observe social spending cuts when we should—based on IMF proclamations—expect social spending to be stable or expand in real terms, then either the IMF’s projections are off the mark or the projections do not devote adequate attention to sheltering the areas of spending that they claim to promote. In either case, the implication for the borrower is the same: constrained fiscal space for social expenditures.

After outlining our data collection approach, our analysis proceeds in three steps. First, we document the return of austerity—that is, rapid and extensive fiscal consolidation targets—in the conditionality attached to IMF loans. This is already alarming for public health and social cohesion, given the evidence that these policy domains are commonly at risk from budget cuts [34–38]. Second, relying on policy documents and official statements, we review the evolving engagement of the IMF with health and social protection issues in its operations. In particular, the organization has now turned ‘social spending floors’ into the spearhead of its new approach, intended to ensure that such spending does not sink below a certain level over the course of lending programs. Third, we present extensive new evidence on the performance of these floors in the IMF’s post-pandemic lending.

## Methods

To initially assess the scale of austerity, we collected government expenditure data for each country from the IMF’s October 2023 World Economic Outlook report for the 2010–2022 period [39]. We then focus on how the IMF has affected the social protection policies of low- and middle-income countries that turned to the IMF for financial support in the 2020–2022 period; that is, after the adoption of the 2019 Strategy for IMF Engagement on Social Spending. We only include the IMF’s long-term lending instruments, the Extended Credit Facility (ECF) and Extended Fund Facility (EFF), as these enable us to trace how social spending and budget balance conditions evolve over time. These loans have a duration of three to four years and make up the majority of IMF programs since 2020.

As Table 1 shows, we collected relevant documentation for 27 long-term loans that were approved in the 2020–2022 period and then collected all available data from their reviews as of 31 August 2023, the endpoint of data collection. This offered a corpus of 101 documents, from which we extracted detailed information on the evolution of social spending and budget balance conditions. In particular, we captured the initial inclusion of such conditions, their subsequent evolution over the course of program reviews, and their implementation status. Importantly, the definitions of social spending floors vary from country to country. For this reason, we extracted the precise definitions available in IMF documentation, available in the Appendix. To enable comparison across countries, we also collected supplementary data on current expenditures to convert condition values from nominal local currency into a share of total spending. Overall, the loans agreed upon between 2020 and 2022 had completed an average of four reviews. Only Sudan and Egypt’s programs had not been reviewed since the initial agreement.

In relation to the IMF’s social spending floors, there is no single way to evaluate whether they are living up to their promise. We seek to tackle this question without resorting to the IMF’s preferred evaluation standards, both for their conceptual shortcomings outlined above and because comparable data for the most recent years covered here are not yet available. Instead, we assess these floors on their own merits, using data solely drawn from IMF loan agreements. To do so, we focus on the stated ambition of spending floors, how these ambitions evolve over the life course of a lending program, whether floors are implemented, and by what margin. If the IMF’s efforts to bolster social spending are sincere, we should expect to see ambitious social spending floors (rather than meagre definitions covering very limited social spending areas), that expand over time (to reflect a progressive increase in ambition), that are implemented by countries (thus reflecting the floors’ appropriate integration into lending program design), and that are generally exceeded (to avoid floors becoming de facto ceilings). We turn to each of these questions next.

**Table 1:** Countries, program, and reviews collected

Country	Date	Program	Initial approval	Review					
				1	2	3	4	5	6
Afghanistan	November 06, 2020	ECF	✓	✓					
Argentina	March 25, 2022	EFF	✓	✓	✓	✓	✓	*✓*	*✓*
Barbados	December 07, 2022	EFF	✓	✓					
Benin	July 08, 2022	EFF & ECF	✓	✓	✓				
Cabo Verde	June 15, 2022	ECF	✓	✓	✓				
Cameroon	July 29, 2021	EFF & ECF	✓	✓	✓	✓	✓		
Chad	December 10, 2021	ECF	✓	*✓*	*✓*				
Congo, Dem Rep	July 15, 2021	ECF	✓	✓	✓	✓	✓		
Congo, Rep	January 21, 2022	ECF	✓	✓	✓	✓			
Costa Rica	March 01, 2021	EFF	✓	✓	✓	✓	✓		
Ecuador	September 30, 2020	EFF	✓	✓	*✓*	*✓*	*✓*	*✓*	✓
Egypt	December 16, 2022	EFF	✓						
Gabon	July 28, 2021	EFF	✓	*✓*	*✓*				
Gambia	March 23, 2020	ECF	✓	✓	✓	✓	✓	✓	✓
Jordan	March 25, 2020	EFF	✓	✓	✓	✓	✓	✓	✓
Kenya	April 02, 2021	EFF & ECF	✓	✓	✓	✓	✓	✓	
Madagascar	March 29, 2021	ECF	✓	✓	✓	✓	✓		
Moldova	December 20, 2021	EFF & ECF	✓	✓	✓	✓			
Mozambique	May 09, 2022	ECF	n/a	✓	✓				
Nepal	January 12, 2022	ECF	✓	*✓*	*✓*				
Niger	December 08, 2021	ECF	✓	✓	✓	✓			
Somalia	March 25, 2020	ECF	✓	✓	*✓*	*✓*	✓	✓	
Sudan	June 29, 2021	ECF	✓						
Suriname	December 22, 2021	EFF	✓	✓	✓				
Tanzania	July 18, 2022	ECF	✓	✓					
Uganda	June 28, 2021	ECF	✓	✓	*✓*	*✓*	✓		
Zambia	August 31, 2022	ECF	✓	✓					

ECF stands for Extended Credit Facility, and EFF for Extended Fund Facility

## Results

### The return of austerity in the IMF’s prescriptions

**Fig. 2:**
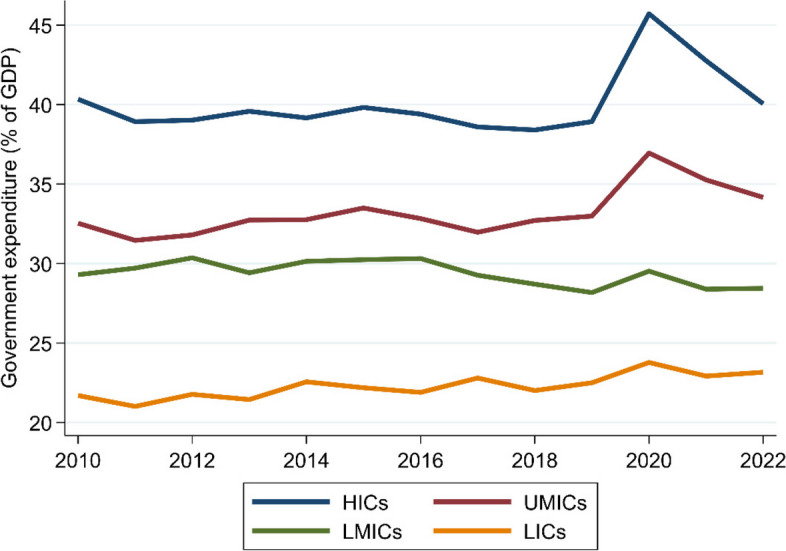
Government expenditure in low, lower-middle, upper-middle, and high income countries. Data source: [39]

Against the dire global economic backdrop described above, budget cuts are increasingly prevalent across the world. In Fig. 2, we present data on government expenditure as a share of GDP between 2010 and 2022, dividing countries into high income (HIC), upper-middle income (UMIC), lower-middle income (LMIC), and low income (LIC) groups, following the World Bank 2020 classification. High-income countries had been experiencing public expenditure declines by the time COVID-19 emerged, linked to budget cuts introduced in the aftermath of the global financial crisis. Once the pandemic was in full swing, these countries increased year-on-year government spending by 6.8% of GDP. Subsequently, these expenditures have experienced a year-on-year decline since 2020, and are expected to reach pre-pandemic levels by 2024 [40]. For upper-middle income countries, public spending had stagnated prior to the pandemic. These countries increased government spending by approximately 4.0% of GDP in 2020, and, despite scaling back spending, are expected to remain slightly above their pre-pandemic levels. In lower-middle income countries, government spending declined through the 2010s and increased by only 1.3% of GDP in 2020, before returning to pre-pandemic levels the following year. And in low income countries, spending stagnated in the 2010s before increasing by 1.3% of GDP in 2020. While spending remained higher than pre-pandemic levels in 2022, this is expected to return to pre-pandemic levels in 2023.

But how does fiscal austerity manifest in the context of IMF programs? There were 21 countries that participated in a long-term IMF loan program that also had data on budget balance conditions available for at least two years of the program, reported in Table 2. To examine the impact of these budget balance conditions, we first need to adjust the second-year figures for inflation, as this meaningfully alters the purchasing power of public expenditure. After making these calculations (based on the IMF’s own GDP deflator data), we find that 15 countries experienced a shrinking of fiscal space between the first and second year of the program. For example, Costa Rica’s central government faced a floor on the cash primary balance of -700 billion colones in 2021 (a fiscal deficit), which was in turn set at 271 billion inflation-adjusted colones in 2022 (a fiscal surplus), representing a 139% shrinkage of fiscal space. In Afghanistan, the operating budget deficit was programed at 154 billion afghani in 2020, but was reduced to 103 billion inflation-adjusted afghani in 2021, a 33% decline in fiscal space.

**Table 2:** Budget balance conditions in recent IMF programs

	Program initiation year	Budget balance condition	Year 1	Year 2	Inflation	Year 2, inflation adjusted	Percentage change, inflation adjusted
Argentina	2022	Floor	-2017.2	-3286.5	112.4	-1547.3	-23%
Benin	2022	Floor	-135.7	-13.1	3.3	-12.7	-91%
Cabo Verde	2022	Floor	-7.8	-6.3	4.5	-6.0	-22%
Cameroon	2021	Floor	-1078.0	-1083.0	5.7	-1024.6	-5%
Chad	2021	Floor	-440.0	-325.0	10.3	-294.7	-33%
Congo, D.R.	2021	Floor	-232.0	-2735.0	6.3	-2572.9	1009%
Congo, Rep.	2022	Floor	-506.0	-523.0	-4.5	-547.6	8%
Costa Rica	2021	Floor	-700.0	287.0	5.8	271.3	-139%
Ecuador	2020	Floor	-3.9	-4.1	2.6	-4.0	2%
Gabon	2021	Floor	-458.9	-470.8	20.6	-390.4	-15%
Kenya	2021	Floor	-507.8	-421.2	6	-397.4	-22%
Madagascar	2021	Floor	-1426.0	-888.0	7	-829.9	-42%
Mozambique	2022	Floor	2.7	7.5	8.1	6.9	157%
Niger	2021	Floor	-484.6	-553.2	3.9	-532.4	10%
Sudan	2021	Floor	-476.0	-296.0	41.5	-209.2	-56%
Suriname	2021	Floor	0.3	3.0	45.6	2.1	518%
Uganda	2021	Floor	-10754.0	-6808.0	8.2	-6292.1	-41%
Zambia	2022	Floor	-8.5	1.1	12.1	0.9	-111%
Afghanistan	2020	Ceiling	154.0	108.0	4.4	103.4	-33%
Jordan	2020	Ceiling	1.8	1.6	1.3	1.6	-10%
Nepal	2022	Ceiling	296.3	256.5	6.3	241.3	-19%

Budget balance values expressed as billions of national currency. Inflation-adjusted values were calculated using the GDP deflator from IMF loan agreements. Kenya, Nepal, and Uganda use end-fiscal year rather than end-calendar year figures. Negative values for percentage changes indicate fiscal austerity for both floors and ceilings. *Source *Authors, drawing on IMF loan documentation

In short, these findings reveal that budget cuts keep forming a core part of the IMF’s policy requests of borrowing countries. Such a development can hamper attempts of these countries to meaningfully invest in their social protection infrastructures, including health. In turn, this sets the stage for a vicious cycle documented in previous crises: cutting the availability of and access to public services at a time of heightened need for social protection policies limits the ability of individuals to respond to economic dislocations, and ultimately undermines economic recovery [17, 20–22, 34–36, 41–43]. 

### The performance of social spending floors in the IMF’s post-pandemic lending

How ambitious are the IMF’s spending floors in terms of current public expenditures? Table 3 presents the relationship between the scope of individual social spending floors and their magnitude. Recall that each social spending floor is separately defined for each country, in consultations with the government. This means that social spending floors are *not* comparable across countries, but only within countries over the duration of the IMF loan, provided the definitions have not changed, as was the case in Afghanistan and Gambia.

One might expect more expansive definitions (that is, those that encompass more areas of spending) to be associated with larger spending floors covering a larger share of current expenditure. The evidence broadly reflects this. For example, countries with less encompassing floors—like Nepal’s, which only covers spending on a child grant—had floors accounting for merely 0.5% of current expenditures. On the other extreme is the case of Tanzania, whose social spending floors cover all spending for health, education, social safety nets, cash transfers, water and sanitation, agricultural inputs, and rural infrastructures, thus adding up to more than half of the country’s current expenditures. This evidence already reveals wide variation between countries, thereby rendering cross-country comparisons of the magnitude of social spending floors of limited utility. Even so, the fact that six IMF loans contain floors that are under 5% of current expenditures suggests that the amount of actual social spending that is purportedly protected is too low. Many vital social policies and public services are excluded and may well face major cuts.

**Table 3:** Social spending floors as share of current spending (initial end-of-year floor)

	Country	As % of current spending
More limited	Nepal	0.5
definition	Congo, Dem. Rep.	0.5
	Barbados	0.8
	Mozambique	1.8
	Gabon	3.9
	Argentina	4.8
	Egypt	5.3
	Suriname	6.7
	Niger	9.0
	Jordan	9.5
	Sudan	10.3
	Madagascar	11.0
	Benin	12.4
	Kenya	15.7
	Congo, Rep.	22.7
	Uganda	24.5
	Cabo Verde	27.2
	Afghanistan	27.3
	Zambia	29.4
	Chad	32.4
	Gambia	33.2
	Moldova	34.2
More expansive	Cameroon	37.1
definition	Tanzania	52.9

Full definition of each country’s floor is presented in the Appendix. Data on current expenditures are drawn from IMF loan agreements and are available in the supplementary data file. *Source* Authors, drawing on IMF loan documentation

**Fig. 3:**
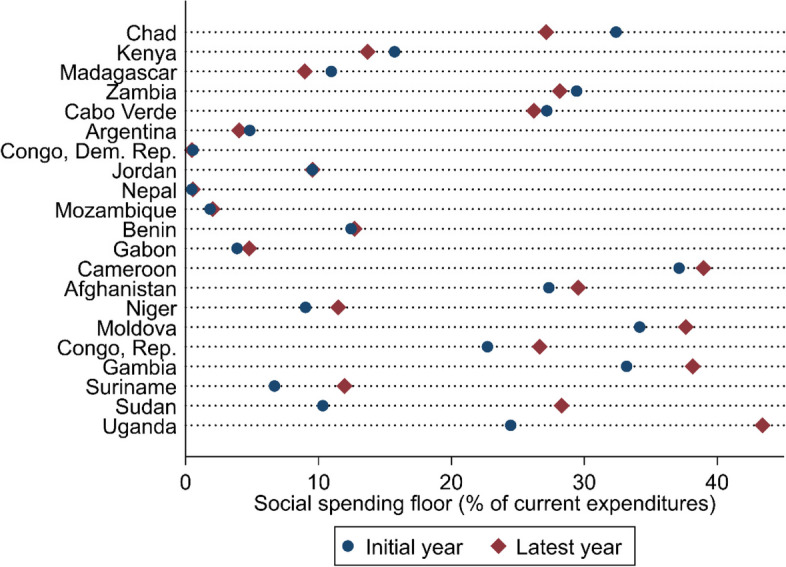
The evolution of social spending floors. Data source: Authors, based on IMF loan documentation

As cross-country comparisons of the social spending floor magnitude are of limited analytical merit due to the varying definitions, we instead ask how social spending floors have evolved over time within individual IMF lending agreements. Figure 3 compares end-of-year floors for the initial IMF program-year with corresponding data for the latest available IMF program-year. For example, it compares Chad’s 2021 social spending floor with that for 2023, both drawn from the conditionality attached to the IMF program. Such a comparison captures the ambition of these floors, as a proxy for the IMF’s commitment to supporting social policies. If social spending floors are meaningfully ambitious, we should expect an increase over time.

The findings presented in Fig. 3 are ordered by the difference between latest-year social spending floor levels with the initial year of the program. Instances where the red diamond is on the right of the blue circle are revealing of non-trivial increases in spending commitments of over 0.5% points encompassed by these floors. Yet, such is the case in only ten out of 21 IMF loans. And in some instances, this is an artifact of changing definitions. A total of five countries experienced only marginal changes to their floors—a ± 0.5% point difference as a share of current expenditures—and are represented by a red diamond placed on-top of the blue circle. More alarmingly, in Argentina, Cabo Verde, Chad, Kenya, Madagascar, and Zambia, social spending floors as a share of current expenditures decreased by over 0.5% points over the course of the IMF loan. These are substantial decreases considering the developmental needs of these countries and that current expenditures—the denominator—may be decreasing in these contexts (as implied by Table 2), and cannot be explained by changing definitions of floors, as they all remain unaltered across the evolution of IMF programs in these countries. This suggests austerity measures are directly eating into social spending.

Are IMF social spending floors implemented? As shown in Table 4, approximately one in three social spending floor conditions were not implemented (37 out of the 117 conditions with implementation data available). The Democratic Republic of Congo, the Republic of Congo, Madagascar, and Mozambique stand out as especially poor performers, meeting none or only one of their spending floors. Implementing these spending floors cannot be seen in isolation, but in the context of the broader fiscal conditions of countries, as overly restrictive budgetary targets may limit funds available for social spending. To this end, we also compare implementation of floors with progress in meeting the IMF’s budget balance conditions. We found 234 budget-related conditions, for which 123 had implementation data available. In total, 81% (100 of 123) were implemented. This higher rate suggests that some countries might be failing to implement social spending floors because of the fiscal efforts to meet budget targets, which—if not implemented—can hold up disbursements of loan tranches. For several countries, budget balance conditions were adhered to, while social spending floor conditions were often unmet. For example, Uganda met all its budget balance conditions but only half of its social spending floors.

**Table 4:** Implementation rates for social spending floors & budget balance conditions, 2020-23

	Social spending floors			Budget balance conditions (or similar)		
Country	Total	of which implementation data available	of which implemented	Total	of which implementation data available	of which implemented
Afghanistan	5	1	0	5	2	2
Argentina	10	6	5	10	6	4
Barbados	6	2	2	6	2	2
Benin	9	3	3	9	3	3
Cabo Verde	9	3	2	9	3	3
Cameroon	12	8	6	12	8	8
Chad	9	3	2	9	3	0
Congo, Dem. Rep.	12	8	0	12	8	6
Congo, Rep.	10	3	1	10	3	2
Costa Rica	0	n/a	n/a	12	8	8
Ecuador	6	6	5	6	6	5
Egypt	3	0	n/a	3	0	n/a
Gabon	10	5	4	10	5	4
Gambia	12	12	11	0	n/a	n/a
Jordan	15	12	12	15	12	10
Kenya	8	5	3	8	5	5
Madagascar	12	9	1	12	9	6
Moldova	8	4	4	9	4	4
Mozambique	8	2	0	8	1	0
Nepal	3	1	1	4	2	2
Niger	11	6	6	11	6	6
Somalia	0	n/a	n/a	12	9	7
Sudan	4	0	n/a	4	0	n/a
Suriname	10	4	2	10	5	2
Tanzania	6	2	2	6	2	1
Uganda	14	8	4	14	8	8
Zambia	8	2	2	8	3	2
*TOTAL*	*220*	*115*	*78*	*234*	*123*	*100*

Afghanistan’s program was cancelled in end-2022; the data presented here cover only 2020 and 2021. For Ecuador, we present implementation data on the condition for household coverage of social assistance measures, which is functionally similar to a social spending floor. *Source *Authors, drawing on IMF loan documentation

But implementation rates alone inevitably offer an incomplete picture. By what margins have countries missed or met their social spending floors? Table 5 reveals that in 11 country-years governments undershot their spending floors, while in 21 instances they exceeded them. In particular, in six instances governments’ social expenditure was over 10% less than what was specified in the IMF-designed floors. But even cases of successful implementation reveal that in many instances (9 out of 21), countries met their social spending floors by a margin of less than 10%. These show that social spending floors—a minimum threshold that public spending on specified social policies needs to meet—are, in practice, acting as ceilings.

**Table 5:** Margins for meeting social spending floors at end-year

Country	Year of floor	Implementation margin
Madagascar	2021	-41.7%
Congo, Rep.	2022	-23.2%
Congo, Dem. Rep.	2022	-22.1%
Congo, Dem. Rep.	2021	-19.0%
Madagascar	2022	-15.2%
Suriname	2021	-13.8%
Chad	2021	-6.7%
Uganda	2022	-5.1%
Cabo Verde	2022	-3.1%
Mozambique	2022	-1.7%
Uganda	2021	-0.1%
Gabon	2021	0%
Afghanistan	2020	0.6%
Argentina	2022	0.8%
Cameroon	2021	1.4%
Kenya	2021	3.3%
Gambia	2021	7.9%
Kenya	2022	8.8%
Jordan	2021	9.0%
Zambia	2022	9.3%
Jordan	2020	9.8%
Niger	2022	13.9%
Suriname	2022	15.8%
Tanzania	2022	18.2%
Gambia	2020	24.6%
Benin	2022	27.4%
Moldova	2022	29.9%
Jordan	2022	30.7%
Barbados	2022	30.9%
Nepal	2022	31.8%
Cameroon	2022	48.4%
Gambia	2022	59.0%
Niger	2021	226.3%

Calendar year is used for all countries except Kenya, Uganda, Tanzania, Barbados, Egypt, and Nepal, where fiscal year is used. *Source* Authors, drawing on IMF loan documentation

## Discussion and conclusions: what way forward for the IMF and social protection?

Our findings reveal cause for concern. Austerity is on the rise, with most IMF borrowers being mandated to substantially cut public spending. To preempt such measures from translating into major cuts to social protection policies, the IMF’s social spending floor approach provides only limited utility: these floors are often set too low and are only implemented two-thirds of the time. Ultimately, this approach remains wedded to ideas of targeted social protection, as the explicit aim of floors is to shelter vulnerable communities from the effects of budget cuts. Thus, at best, social spending floors act as damage control for the painful budget cuts: they are instruments of social amelioration, underpinned by principles of targeted assistance for highly disadvantaged groups. At worst, these floors can be seen as a distraction from the urgent policy work necessary to ensure that IMF programs do not jeopardize health and social protection policies.

Overall, our findings suggest that the social spending floor approach is opaque and inadequate. Decisions on the levels and content of floors vary widely from country to country and can be easily tweaked by changing definitions to show compliance. In addition, such floors can be a policy distraction: instead of starting from a principled and evidence-based position on the social spending needs of the borrowing country, social spending floors haphazardly aggregate disparate areas so that the IMF can signal that its modus operandi now addresses long-standing criticisms. Even so, their poor record in implementation suggests that many governments do not meaningfully commit to meeting them.

In reaching these conclusions, we point out two methodological limitations of our work. Our analysis draws exclusively on (a) highly aggregated data that (b) is available exclusively within IMF loan agreements. The primary advantage of using this data is availability for the latest period, compared to health expenditure data generated by the World Health Organization, which was only accessible up to 2021 at the time of publication. Yet, as we have seen, the social spending floor figures between countries are not comparable, as they include a range of disparate policy areas (sometimes as little as only a cash transfer program, while in other cases as much as all spending in health, education, water, sanitation, agriculture, and infrastructure development). Future research can juxtapose our findings to the actual social spending trajectories of low- and middle-income countries, disaggregated into fine-grained spending areas. This can serve as the ultimate assessment of the merits or failures of the social spending floor approach.

The only route for durably reducing inequalities in the Global South is through universal social protection policies [44–47]. In this endeavor, the IMF’s Social Spending Floor initiative is a poor guide. As a more promising alternative, the International Labor Organization has pioneered the Social Protection Floor approach, which promotes universal access to social services and social security transfers across the life-course. This approach rests on a nationally-determined minimum that countries must guarantee all residents in areas (such as access to essential healthcare and income security), and requires ‘coherent policies within and across the social, economic and health sectors […that can break] the mutual linkages between ill health, poverty and other vulnerabilities’ [48].

This focus on universal provision marks a decisive shift away from the IMF’s preference for targeting of policies to vulnerable groups. A common defense of the IMF position is that many Global South countries cannot afford expansive provision of welfare to the population. Yet, ‘universalism’ need not mean that everyone is eligible for an equal amount of services. Instead, principles of ‘proportionate universalism’ can be applied to safeguard the universal nature of social provision, while still enabling a degree of selectivity in interventions. This means the ‘scale and intensity …[of interventions must be] proportionate to the level of disadvantage’ [49].

A key barrier to pursuing such a universalist approach to social protection are the excessively high debt burdens they are currently facing, as discussed in the Introduction. In practice, this means that progress will likely be coupled with comprehensive responses to resolving the ongoing debt problems. During the COVID-19 pandemic, some early steps in that direction were taken through the Debt Service Suspension Initiative which sought to help participating low and lower-middle income countries concentrate spending on the pandemic response by suspending debt service owed to bilateral and multilateral creditors. However, discussions over the so-called Common Framework—a coordinated and comprehensive approach to dealing with debt problems of highly-indebted countries—has failed to create any consensus among traditional bilateral creditors (known as the Paris Club), emergent major bilateral creditors (most notably, China), and the private sector [50].

Ultimately, the IMF’s policy advice to countries needs to be informed by its mandate of facilitating ‘national and international prosperity’ [51]. This means abandoning ceremonial reforms and short-termism, and instead committing to universalist policies that can help countries both meet their economic objectives (like macroeconomic stabilization and equitable growth) and their social development priorities (like improving health and welfare) [38]. In this path, the organization can draw on the recent experience of the World Bank, which in 2022 identified universal social protection as a key priority in its lending operations [52]. To be sure, the IMF has a mandate to promote macroeconomic and financial stability, and not for social development. But, as we have shown in this article, even within this mandate, there is ample scope to ensure that its lending programs facilitate the achievement of universal social protection objectives and that adequate fiscal space is created with that end goal.

## Data Availability

The dataset generated and analysed for the current study are available in the Harvard Dataverse repository, 10.7910/DVN/KVVFV1, and on www.imfmonitor.org.

## Appendix

**Table A1:** Definitions of Social Spending Floors in EFF and ECF loans agreed in 2020-2023

*Country*	*Social spending floors encompass…*
Nepal	Child grant spending. The child grant reaches vulnerable households, is implemented by the federal government, and is monitorable in a timely way.
Congo, Dem. Rep.	The sum of: (i) Reproductive, Maternal, Neonatal, Child and Adolescent Health and primary health care spending; (ii) disbursement of Gavi-supported vaccine co-financing and traditional vaccines procurement, and (iii) disbursement of TB/Malaria/HIV/AIDS co-financing.
Mozambique	Transfers to Instituto Nacional de Acçao Social from the budget (through the treasury single account, i.e., not including transfers to INAS through project grants or project loans from external partners).
Argentina	The cumulative sum of all federal government spending (both recurrent and capital) on the following social assistance programs: (i) Asignación Universal para Protección Social, which includes the following sub-programs: Asignación Universal por Hijo, Asignación por Embarazo, and Ayuda Escolar Anual; (ii) Tarjeta Alimentar, and (iii) Progresar
Gabon	The sum of: (i) social services relating to social safety nets, free childbirth coverage, SAMU Social and seniors; (ii) legal assistance; (iii) the costs of the electrification program and hydraulic installations intended for rural areas without access to public water and electricity network; and (iv) the special solidarity contribution allocated to economically weak Gabonese.
Suriname	All the spending of the Ministry of Social Affairs and Public Housing on social protection programs, including the following cash transfer programs: General old-age pension; General Child benefit; Financial assistance for persons with disabilities; and Financial assistance for weak households.
Congo, Rep.	Public expenditure in priority social sectors deemed to be conducive to poverty reduction.
Barbados	Expenditures incurred by the central government on the following plans and programs, excluding operating expenditure, that are intended to have a positive impact on education, health, social protection, housing and community services and recreational activities: Welfare Department spending including cash transfers and assistance for house rents, utilities, food, and education to the poor and vulnerable; Child Care Board spending on protection of vulnerable children; Youth Entrepreneurship Scheme assisting jobless youth to start own businesses; Strengthening Human and Social Development program targeting the unemployed and vulnerable families and youth; Alternative Care for the Elderly program targeting the elderly transferred to private care; and Provision of medication to HIV patients.
Egypt	Spending related to the budget of the Ministry of Health and the Ministry of Social Solidarity.
Jordan	The sum of: (i) non-wage components of the education and health sectors’ current expenditure envelope, including all spending directly related to efforts to prevent, detect, control, treat and/or contain the spread of COVID-19 spending; (ii) the National Aid Fund’s and other entities’ social protection programs; and (iii) the school feeding program.
Sudan	Spending on direct cash transfer, education, health, and training.
Kenya	The sum of: (i) cash transfers to orphans and vulnerable children; (ii) cash transfers to elderly persons; (iii) cash transfers to persons with severe disabilities; (iv) free primary education expenditure, free secondary education expenditure, school food and sanitary programs; and (v) free maternal healthcare, universal health coverage, health insurance subsidy for targeted categories (i.e., orphans, vulnerable children, elderly, and people with disabilities), and spending for vaccination and immunization.
Cabo Verde	expenditures incurred by the central government on the plans and programs that are intended to have a positive impact on education, health, and social protection, excluding the wages and salaries component.
Madagascar	The sum of budget allocations to the Ministries of Health, Education, Population and Water, excluding salaries and externally financed investment.
Benin	Expenditure executed from the State budget (from both domestic and external resources), excluding salary expenditure and relating mainly to public interventions in the areas of education, health and nutrition, the establishment of social safety nets, access to electricity, water and sanitation, microfinance (small and medium enterprises), as well as security and to civil protection. Priority social spending (PSE) is very selective and captures only spending that directly reduces poverty.
Uganda	All spending in health, education, and social development (excl. external financing).
The Gambia	Initial loan approval: Expenditures financed out of The Gambia Local Fund (GLF) on the following areas: Agriculture and Natural Resources; Education; Health; Nutrition, Population and HIV-AIDS; Infrastructure Program; Social Fund for Poverty Reduction; Implementation and Monitoring of Poverty Reduction Programs; Support to Cross- Cutting Programs; ICT Research and Development; Decentralization and Local Government Capacity Building; Governance and Civil Service Reform Program.
	Second review: As above, but starting Q1 2021, the poverty-reducing expenditure will include the COVID-19 spending implemented through the COVID-19 project accounts.
Niger	Expenditures from the Government's own resources allocated to the social sectors and those directly benefiting poor households, children, young people and women in vulnerable situations, the elderly, the disabled, victims of armed conflict and trafficking, refugees, or displaced persons and the unemployed.
Afghanistan	Initial loan approval: the sum of pro-poor spending identified in accordance with the Afghanistan National Development Strategy poverty profile by the Ministry of Education, Ministry of Public Health, and Ministry of Labor, Social Affairs, Martyrs, and Disabled.
	First review: the sum of social spending identified in accordance with the Afghanistan National Peace and Development Plan II, by the central government, aiming at benefiting the poor and vulnerable populations in areas of education, healthcare, food and nutrition security, social safety net, pensions for martyrs and disabled, refugees and repatriates, skills development, women empowerment, and pandemic and natural disaster relief, within the central government’s operating and development budget during a fiscal year.
Moldova	Two separate loan conditions: (a) The sum of support for unemployment, the social assistance program (Ajutor Social), as well as the heating allowance during the cold season and the government’s energy poverty policy from the central government budget, and (b) developmental spending undertaken by the general government (Includes health, educational, and infrastructure spending)
Cameroon	The sum of: (i) for the education sector, total expenditure (current and capital) of the Ministries (Basic Education, Secondary Education, and Employment and Vocational Training); (ii) for the health sector, current and capital expenditure of the Ministry of Public Health, including COVID-19 related expenditures; and (iii) for other social sectors, current and capital expenditure of the Ministries of Labor and Social Security, Youth and Civic Education, Social Affairs, and Promotion of Women and Family; (iv) administered price subsidies (fuel at the pump, electricity to households), and (v) expenditures for the Social Safety Net Program.
Zambia	Central government expenditure on the Social Cash Transfer, Food Security Pack, Empowerment Fund (Women and Youth), the Public Welfare Assistance Scheme, Water and Sanitation, budget transfers to the Public Service Pensions Fund, the Health Sector, and the Education Sector
Tanzania	Central government spending (recurrent and development) for education, health, water, social safety nets (including cash transfers through Tanzania’s Social Action Fund -TASAF), rural electrification, agricultural inputs, as well as for upgrading and maintenance of rural roads, including transfers to local governments for health, education, and rural water supply.
Chad	Public spending by the following ministries: (i) National Education and Civic Promotion; (ii) Public Health, including military health services and National Solidarity; (iii) Women, Early Childhood Protection and National Solidarity; (iv) Production, Irrigation and Agricultural Equipment; (v) Livestock and Animal Production; (vi) Environment Water and Sanitation; (vii) Professional Training and small Job Promotion, and (viii) Higher Education.

Sources: Individual IMF loan agreements, edited for conciseness
